# Gait and Equilibrium in Subcortical Vascular Dementia

**DOI:** 10.1155/2011/263507

**Published:** 2011-03-13

**Authors:** Rita Moretti, Paola Torre, Rodolfo M. Antonello, Francesca Esposito, Giuseppe Bellini

**Affiliations:** Medicina Clinica, Ambulatorio Complicanze Internistiche Cerebrali, Dipartimento Universitario Clinici di Scienze Mediche Tecnologiche e Traslazionali, Università Degli Studi di Trieste, Ospedale di Cattinara, Strada Fiume 447, 34149 Trieste, Italy

## Abstract

Subcortical vascular dementia is a clinical entity, widespread, even challenging to diagnose and correctly treat. Patients with this diagnosis are old, frail, often with concomitant pathologies, and therefore, with many drugs in therapy. We tried to diagnose and follow up for three years more than 600 patients. Study subjects were men and women, not bedridden, aged 68–94 years, outpatients, recruited from June, 1st 2007 to June, 1st 2010. We examined them clinically, neurologically, with specific consideration on drug therapies. Our aim has been to define gait and imbalance problem, if eventually coexistent with the pathology of white matter and/or with the worsening of the deterioration. Drug intake interference has been detected and considered.

## 1. Introduction

It has been recently stated [1] that gait disorders are common in the elderly and are related to loss of functional independence and death. Cerebral small vessel disease is related to gait disturbances: in particular, white matter lesions and lacunar infarcts were both independently associated with most gait parameters, in particular with stride length [1]. These authors concluded that white matter lesions in the basal ganglia and internal capsule and limbic areas and lacunar infarcts in the frontal lobe and thalamus were related to a lower velocity [1]. It has been established recently a direct relationship between subcortical vascular lesions and falls, even if works on the matter are scarce [2–4]. Age-related cerebral degenerative changes are coupled therefore with decreased perfusion, usually assumed to be secondary to decreased cerebral metabolic demands [5]. During ageing declines in cerebral tissue densities in gray (polio-araiosis) and white matter (leukoaraiosis) reflect neuronal degenerative changes, which progress concurrent with cerebral perfusion declines. Rates of polio- and leukoaraiosis accelerate geometrically after age 60, correlating with cortical and subcortical atrophy and ventricular enlargement. In particular, leukoaraiosis correlates with advancing age, cerebral atrophy, hypoperfusion of white matter, and cognitive impairments [6]: what is surprising is that leukoaraiosis is detectable in 9–19% of older “normal” subjects but is virtually always present in vascular dementia. Of special interest are the data emerging from the study of Meyer et al. [6]: normative subjects destined for later cognitive decline had excessive leukoaraiosis at study entry, suggesting that leukoaraiosis is, itself, a risk factor for cognitive decline. Vascular dementia refers to a broad category of patients where a multifaceted cognitive decline is attributed to cerebro- or cardio-vascular disease. It has long been known that cognitive deficits may result from a stroke, yet only recently have reports demonstrated that dementia may occurs in approximately one-fourth to one-third of stroke cases [7]. Apart from that dementia is due to single strategic infarcts seated in critical areas, such as hippocampus, thalamus, and caudate nucleus, and to intracranial hematomas or mixed forms, vascular dementia (VaD) is also associated with subcortical ischemic vascular disease caused by small-vessel disease leading to lacunar infarcts and ischemic white matter changes. Only recently, the International Classification of Diseases (10th revision) (ICD-10) criteria for VAD explicitly identified subcortical VaD as a subgroup [8]. Subcortical VaD, therefore, incorporates the old entities “lacunar state” and “Binswanger disease” and relates to small vessel disease and hypoperfusion resulting in focal and diffuse ischemic white matter lesion and incomplete ischemic injury [9, 10]. Two pathophysiological mechanisms, sharing common risk factors, lead to dementia associated with small-artery disease [11]: one possible mechanism is defined by occlusion of an arterial lumen, which conducts to a complete infarct (lacunar infarct) and leads to dementia due to the disruption of a neural pathway. The other mechanism involved is defined by critical stenosis and hypoperfusion of multiple arterioles. This results in widespread areas of incomplete infarction of the deep white matter and consequent functional disruption of neural network. The end stages of the two pathways are labeled as lacunar state and Binswanger syndrome, which, in practice, usually converge. 

Cerebrovascular disease is common to the elderly and can cause a wide spectrum of impairments ranging from mild to severe. Sensitive and specific definitions of cerebrovascular cognitive impairment are hampered by the fact that cerebrovascular disease is not easily linked to cognitive syndromes either clinically or pathologically and the presence of coincident AD is common. In a well-defined population suffering from subcortical vascular dementia (standing from a neurological, clinical, and radiological criteria) we have tried to define gait, balance, and equilibrium alterations, even considering precipitant factors, such as concomitant pathologies and consequent therapies. 

## 2. Subjects and Methods

### 2.1. Methods

#### 2.1.1. Patients

Study subjects were men and women aged 68–94 years, entering in Cognitive Disorder Unit Evaluation of the University of Trieste, with Mini-Mental State Examination (MMSE) scores of at least 14 and satisfying the fourth edition of the Diagnostic and Statistical Manual of Mental Disorders (DSM-IV) for dementia, recruited from June 1st 2007 to June 1st 2010. We have examined 2345 patients, who have been diagnosed as suffering from mild cognitive impairment, Alzheimer's Disease, frontal lobe dementia, Lewy body dementia and Parkinson-dementia complex. 817 patients suffered from vascular dementia, and 171 have been diagnosed as having multi-infarct dementia. To this group belonged 21 patients suffering primarily from brainstem infarction. 646 patients suffered from subcortical vascular dementia: these study subjects satisfied the criteria for probable VaD in accordance with the NINDS-AIREN criteria (Román et al., 1993). A patient was diagnosed as having subcortical VaD (sVaD) when the CT/MRI scan showed moderate to severe ischaemic white matter changes (Erkinjuntti et al., 1999) and at least one lacunar infarct. 

Brain CT scans or MRI images were randomized and assessed independently, after the radiologist's opinion, by neurologists (RM, PT). 

Patients were not included in the study if they showed signs of normal pressure hydrocephalus, previous brain tumours, previous diagnosis of major stroke or brain haemorrhage. We did not include patients with white matter lesions, caused by specific aetiologies, such as multiple sclerosis, brain irradiation, collagen vascular disease, and genetic forms of vascular dementia (such as CADASIL or CARASIL). Patients with previous major psychiatric illness (i.e., schizophrenia, bipolar disorders, psychosis, compulsive-obsessive disorders, etc.) or central nervous system disorders and alcoholism were excluded too. We did not consider a discriminant/exclusion criteria depression, according to different studies (such as [12]), according to the potential correlation to vascular dementia predisposing factor. 

#### 2.1.2. Study Design

This was a prospective cohort study, designed to investigate gait (balance and equilibrium) disorders, behavioural alterations, drug intake of a subcortical dementia population. Study subjects were 646 men and women, diagnosed as previously stated as subcortical vascular dementia (sVaD), not bedridden, aged 68–94 years outpatients, recruited from June, 1st 2007 to June, 1st 2010, who underwent a standardized baseline assessment that included a detailed history, a physical examination, laboratory tests, and psychiatric evaluations. The physical examination included evaluations of pulse rate and rhythm, blood pressure, heart size and sounds, peripheral pulses, retinal vessel and carotid artery evaluation, electrocardiographic evaluation, and chest X-ray. The physical examination was repeated at every visit; electrocardiographic evaluation and laboratory tests were repeated every 12 months. Patients in both groups were allowed to continue any previous therapy (e.g., cholinesterase inhibitors, antihypertensive, antidyslipidemic, antidiabetic drugs). (Table 1). All patients were followed with periodical neurological and neuropsychological examinations. Visits were scheduled to take place every four months. A complete neuropsychological examination was conducted at baseline and every 6 months; every 12 months' results were compared. The trial was conducted in accordance with the Declaration of Helsinki and with the Ethics Guidelines of the Institute. 

#### 2.1.3. Outcome Measures

Global performance was assessed using the Clinical Dementia Rating (Hughes et al., 1982) at every visit. Behavioural symptoms were assessed using the Neuropsychiatric Inventory (NPI) (Cummings et al., 1994) at every visit. Functional activities were assessed by the Barthel index (BI) (Mahoney and Barthel, 1965), and complex activities of daily living were evaluated by the Instrumental activity of Daily living (IADL) (Lawton and Brody, 1969). Mobility problems were assessed by the Tinetti scale for equilibrium/balance and gait (Tinetti, 1986) at every visit. Semiquantitative assessment consisted of the modified Tinetti test with 17 items: 9 for body balance (score 0 to 16) and 8 for gait (score 0 to 12) with a maximum score of 28. Hachinski Ischemic score (HIS) was done at every visit (Hachinski et al., 1975). Patients were registered for their medical intake. 

#### 2.1.4. Statistical Analyses

Statistical analyses were performed using the Statistical Package for the Social Sciences (SPSS, version 16.0). Within-group changes from baseline to 12, 24, and 36 months were tested using the Wilcoxon Signed Ranks test. This was done for the overall scores for each efficacy variable. 

Spearman's rank correlation analyses were performed between behavioral outcome measures, Tinetti scale, global, balance and equilibrium, and BI. Results are presented as mean changes from baseline with standard deviations, and *P* values are presented where appropriate. 

## 3. Results

Among 2345 patients, 817 were diagnosed as having vascular dementia: 171 did have multi-infarct dementia, and 646 patients had subcortical vascular dementia; the latter, 320 men and 326 women, were included in the study (Table 1). Their mean age was 73.45 ± 7.12 years, and they had a mean education level of 9.45 ± 1.19 years. 

The diagnosis was based on historical information and neuropsychological assessment and supported by findings on structural (CT or magnetic resonance) imaging. Subsequent follow-up of subjects has reinforced the clinical diagnoses in all cases. 

Brain CT scans or MRI images were available for all the 646 selected patients; 246 patients did MRI studies, and 456 did CT scans. Obviously, the sensitivity for observing subcortical lesions is greater with MRI compared to CT scans. Though, considering that all the patients went to our unit for a specialist evaluation and eventual follow-up by their general practitioners, it was obvious that they prescribed more easily CT scans rather than MRI imaging. For 400 patients, CT scans, history, neurological, and neuropsychological tests were sensitive and sufficient to make the diagnosis. 56 patients were requested by us to complete their diagnostic route with MRI sequences, in case of not adequate imaging acquisition or not convincing data. 190 patients came to our unit with MRI images, prescribed by their general practitioners or by different specialists who they attended before our visit. Therefore, the patients who did CT/MRI were homogeneously recruited, and no demographical/social/ cultural/clinical difference distinguishes them from each other. 

Two neurologists of the group revised all the imaging, employing the Blennow et al. [13] scale for CT scans and the Scheltens et al. [14] scale for MRI imaging. There was 96.7% interrater agreement for the independent assessment of the scans (kappa = 0.79). 

Twenty four patients died during follow-up; sixteen patients did not have a caregiver, who could guarantee adequate compliance and therefore have been excluded, after the first year from the study. All the other 606 patients (304 men and 302 women) completed the full 36-month study. Patients were allowed to continue any previous therapy (e.g., antihypertensive, antidyslipidemic, antidiabetic drugs) (Table 2). During the follow-up, the patients were prescribed neuroleptics and/or benzodiazepines (Table 3). 

Considering the global health conditions, there was an obvious deterioration in the patients as evidenced by the increase of the daily drug uptake (Table 3).

Main scores obtained by the patients, during the follow-up, have been reported in Table 4 as mean and SD. Particularly, the results obtained in the Tinetti Scale, considered as subscores gait and equilibrium, as said before, and the total score, have been reported in Table 5. According to a Wilcoxon Signed Ranks test, there was a general worsening of the clinical, cognitive, behavioural, and instrumental capacities of the patients observed in the 36 months of follow-up (Table 6). Moreover, there was a dramatic decrease in gait and equilibrium control in the patients during follow-up (Table 7). In particular, we observed a significant decrease in Tinetti global scores (and separate subitems) in the diabetic group (263 patients) and in the cardiopatic patients (149). In particular, as far as 102 patients suffered of the two conditions at the same time, their Tinetti scores are the worst (significantly different from the scores obtained by 162 patients who suffered from diabetes only (*P* < .01) and from the scores done by the 47 patients (*P* < .05) who suffered from cardiac pathologies. 

Spearman's rank correlation analyses indicated that there was a significant correlation between gait scores (total and separately, gait and equilibrium) and Clinical Dementia Rating Scales scores (CDR = 2.13 ± 0.11, *r* = 0.81, *P* < .05 over 12 months; CDR = 2.84 ± 0.4, *r* = 0.83, *P* < .01 over 24 months; CDR = 2.98 ± 0.61, *r* = 0.89, *P* < .01 over 36 months). Spearman's rank correlation analyses indicated that there was a significant correlation between Tinetti gait score and BI over 24 months (BI = 76.84 ± 6.11, *r* = 0.72, *P* < .05), Tinetti equilibrium score and BI over 36 months (BI = 61.17 ± 7.3, *r* = 0.71, *P* < .01), and Tinetti gait score and BI over 36 months (BI = 61.17 ± 7.3, *r* = 0.78, *P* < .01). Moreover, we have found a correlation between NPI scores and Tinetti gait score over 24 months (NPI = 56.84 ± 12.11, *r* = 0.81, *P* < .01), Tinetti equilibrium score and NPI over 36 months (NPI = 66.03 ± 8.81, *r* = 0.87, *P* < .01), and Tinetti gait score and NPI over 36 months (NPI = 66.03 ± 8.81, *r* = 0.78, *P* < .01), and of course, Tinetti total score and NPI over 36 months (NPI = 66.03 ± 8.81, *r* = 0.93, *P* < .01).

Surprisingly, we have found only a correlation between benzodiazepines intake and Tinetti equilibrium score at 12, 24, and 36 months (resp., *r* = 0.77, *P* < .05; *r* = 0.78, *P* < .05; *r* = 0.67, *P* < .05 at 12 months; *r* = 0.81, *P* < .01; *r* = 0.77, *P* < .05; *r* = 0.84, *P* < .01 at 24 months; *r* = 0.81, *P* < .01; *r* = 0.82, *P* < .01; *r* = 0.89, *P* < .01 at 36 months). 

Considering neuroleptics, one should take into account the employment of typical or atypical neuroleptics. Moreover, quetiapine, as an atypical neuroleptic shows less dopamine affinity than olanzapine, which we have examined through the study. Therefore, evaluating separately the results, we have observed the following relationships.

**Typical neuroleptics**

We have found a significant correlation between haloperidol intake and Tinetti equilibrium score at baseline and at 12 months (resp., *r* = 0.61, *P* < .05 and *r* = 0.72, *P* < .05); between haloperidol intake and Tinetti total score at 24 and 36 months (resp., *r* = 0.81, *P* < .01 and *r* = 0.86, *P* < .01); not significant correlation between promazine chloridate intake and Tinetti subscores at baseline and at 12 months; we have found a positive correlation between the equilibrium score of Tinetti test and promazine intake at 24 months (*r* = 0.74, *P* < .05) and a positive correlation between the equilibrium score and the gait score of Tinetti test and promazine intake at 36 months (resp., *r* = 0.79, *P* < .01 and *r* = 0.81, *P* < .01).

**Atypical Neuroleptics**

We have found a significant correlation between olanzapine intake and Tinetti equilibrium score at 12 and at 24 months (resp., *r* = 0.61, *P* < .05; *r* = 0.68, *P* < .05); between olanzapine intake and Tinetti total score at 36 months (resp., *r* = 0.71, *P* < .05); we have found a positive correlation between the equilibrium score of Tinetti test and quetiapine intake at 24 and at 36 months (resp., *r* = 0.77, *P* < .05; *r* = 0.80, *P* < .01). The mean dose of olanzapine remained stable during the 12th up to the 36th months of follow-up (5.1–5.9 mg/day); on the contrary, quetiapine dosage increased during up to 36th months of follow-up (56.9–89.9 mg/day).

## 4. Discussion

Walking is a complex mechanism, based on motor control, step rhythm, muscular activation and dysactivation, motor adjustment, attention, perception, and so on. Spinal and brainstem activation, which seem to be fundamental for quadrupeds, in humans depend more on cortical and subcortical inputs in humans [15]. Therefore, it has been hypothesized that the association between step length variability and basal ganglia integrity is consistent with our knowledge of the role played by the basal ganglia in controlling mobility, because of their functional connection with the other motor areas, the basal ganglia control motor coordination and rhythmicity [16, 17]. As well pointed off in the study by Rosano et al. [16], the basal ganglia exerts a compensatory mechanism in gait control, in particular in gait variability. In their study, subclinical brain vascular abnormalities were measured on brain MRI as infarcts and white matter hyperintensities. They demonstrated that greater variability of step length was associated with greater prevalence of infarcts and greater white matter hyperintensities severity, independent of age, gender, and cognitive function [16] To implement these data, another study by Carboncini et al. [15] demonstrated that higher level gait disorders are a common gait alteration in older people and that gait disorders can occur in a neurological syndrome named as leukoaraiosis. Their perfusional functional cerebral SPECT demonstrated that the rCBF increase induced by the treadmill test (functional versus baseline), bilaterally in the medial frontal gyrus and in the anterior lobes of the cerebellum, resulted significantly lower in patients with gait apraxia versus those without it, all of them having diffused chronic ischemic lesions within white matter [15]. Data have been clinically confirmed by other works by the LADIS group [4], by Srikanth et al. [18], and by Masdeu and Wolfson [19]. 

The results of our study might be considered at first instance as not unexpected and not completely novel. Though, trying to evaluate them, step by step, we could consider that they are the first results on gait and equilibrium in a selected subcortical vascular dementia population, with a 36-month follow-up. Our prospective evidence demonstrates that in a selected subcortical vascular dementia group, diagnosed either accomplishing neurological or neuroimaging criteria, there is major evidence of significant gait and equilibrium problems, from the beginning of the observational period, and they become worse during the three years of follow-up. A clear point should be made standing from the firmly exclusion criteria of brainstem lesions, in order to eliminate potential confounding effect of specific ischemic lesions on gait and equilibrium. Therefore, gait and balance alterations observed in this population should be considered as a result of subcortical widespread damage, and not of other problems, such as pharmacological effects or superimposing comorbidities; during follow-up, the gait abnormalities are stressed by the general worsening, the decrease of behavioural control, and the consequent neuroleptic and benzodiazepine intake. 

Therefore, it might be concluded that subcortical lesions, per se, cause imbalance and gait alteration: we might hypothesize that this subcortical hypoperfusion might interrupt long loop reflexes of deep white motor tracts and descending motor fibers arising from medial cortical areas (see data and literature in: [3, 20]); moreover, subcortical vascular lesions interest fibres connecting frontal cortex and subcortical structures, which are responsible for motivation, executive function, planning, and attention too (see in particular frontal eye fields). It has been suggested (see data and literature in [20]) that the basal ganglia maintains cortically selected motor set in the supplementary motor area and provides internal cues to the supplementary motor area in order to enable each submovement to be correctly linked together Iansek et al. [21]. It has been demonstrated that attention problems, in particular of shift attention and focused attention, decrease the performance of gait in Parkinsonian patients with freezing of gait [22–24]. 

These data seem to be in accordance with what we observed, at baseline: subcortical demented patients might show gait problems just for their vascular altered white substance. However, it must be considered that our subcortical vascular population is not a mathematical model. The follow-up of the pathology is tightly connected with the general worsening of health condition, which conduct to a deterioration of gait in different ways: due to general strength diminishment, bad tolerance to physical effort (such as in cardiac pathologies), or due to an evident alteration of glucose metabolism, with consequent peripheral alterations (polyneuropathy, peripheral arterials alterations), and/or bad resistance to muscular efforts. 

On the other hand, the evolution of subcortical dementia leads to behavioural alteration, as well as cognitive impairment worsening (in particular of executive function and frontal focusing). The former imposes a specific therapy, based on sedative and neuroleptic drugs. They might be related to hypotonia, altered balance, reduced postural reflex, static imbalance, parkinsonism, and so forth. All these aspects worsen the gait profile, as documented by our results. Moreover, in an old and sensitive population, like the one considered in this study even the generally considered safest drug, such as atypical neuroleptics (and among them, even the less dopamine receptor affine, as quetiapine), might cause unexpected but significant alteration of balance and gait, probably due to the general favourable ground determined by the primary brain condition.

All these facts considering subcortical vascular dementia may be associated with gait and balance alteration *per* *se*; its worsening and the pharmacological choices, imposed by the evolution of the pathology, should be considered by the clinicians in order to prevent major gait alteration, falls, and postural imbalance, or at least, to reduce their consequence in a real frail population. 

## Figures and Tables

**Table 1:** Baseline characteristics of patients.

Age in years (mean ± SD)	73.45 ± 7.12
Gender (male/female)	304/302
Education level in years (mean ± SD)	9.45 ± 1.1

Concomitant illnesses (N.+ % patients)	
Essential hypertension	189 (31.18%)
Diabetes mellitus, type 2	236 (38.94%)
Ischaemic cardiopathy/valvular failure/arrythmias	149 (24.58%)
Chronic obstructive bronchopathy	179 (29.53%)
Neoplasia	23 (3.79%)

**Table 2:** A synopsis of the drugs employed by the two groups of patients at baseline.

Drug utilization mg/day	Baseline	12 months	24 months	36 months
ACE inhibitors	54 patients	89 patients	94 patients	164 patients
Enalapril, mean ( ± SD) dose	27. 4 ± 4.3	25.56 ± 3.65	25.56 ± 3.65	25.56 ± 3.65
Ramipril, mean ( ± SD) dose	3.41 ± 2.8	3.1 ± 1.89	3.1 ± 1.89	3.1 ± 1.89
Quinapril, mean ( ± SD) dose	5.11 ± 1.1	7.41 ± 0.8	7.6 ± 1.4	9.41 ± 1.8

Sartan	78 patients	67 patients	89 patients	102 patients
Losartan, mean ( ± SD) dose,	45.56 ± 23.54	49.67 ± 21.23	49.67 ± 21.23	49.67 ± 21.23
Telmisartan, mean ( ± SD) dose	43.12 ± 12.23	45.12 ± 1.45	40.2 ± 9.7	41.3 ± 9.6
Candesartan, mean ( ± SD) dose	16. 2 ± 2.32	16. 7 ± 6.16	12. 2 ± 5.65	14. 2 ± 4.32

Calcium antagonists	84 patients	93 patients	123 patients	153 patients
Amlodipine, mean ( ± SD) dose	6.0 ± 4.10	5.6 ± 2.34	5.6 ± 2.34	5.6 ± 2.34
Felodipine, mean ( ± SD) dose	7.5 ± 2.21	6.9 ± 3.5	6.9 ± 3.5	6.9 ± 3.5
Lacidipine, means ( ± SD) dose	4.5 ± 1.34	7.5 ± 0.21	7.67 ± 1.3	5.56 ± 2.7

Antidiabetic medication	86 patients	112 patients	98 patients	105 patients
Glimepiride, mean ( ± SD) dose	1.0 ± 0.34	1.5 ± 0.56	1.43 ± 0.87	2.5 ± 0.12
Glibenclamide, mean ( ± SD) dose	5.5 ± 2.12	6.7 ± 1.43	7.5 ± 2.12	7.7 ± 3.5
Repaglinide, mean ( ± SD) dose	2.5 ± 0.34	2.65 ± 1.34	3.0 ± 1.31	4.0 ± 0.3
Metformina, mean ( ± SD) dose	950.5 ± 43.30	1200.5 ± 140.3	950.7 ± 443.3	1150.5 ± 153.3

Antiplatelet drugs	111 patients	149 patients	210 patients	249 patients
Ticlopidine, mean ( ± SD) dose	250 mg, b.i.d	250 mg b.i.d	250 mg b.i.d	250 mg b.i.d

ASA	117 patients	224 patients	326 patients	378 patients
Mean ( ± SD) dose	300	300	300	300

Digoxin	23 patients	25 patients	25 patients	25 patients
Mean ( ± SD) dose	0.23 ± 0.12	0.22 ± 0.13	0.22 ± 0.13	0.22 ± 0.13

Loop diuretics	53 patients	87 patients	78 patients	96 patients
Furosemide	29.45 ± 7.89	28.56 ± 8.92	28.56 ± 8.92	28.56 ± 8.92

Thiazide diuretics	65 patients	87 patients	93 patients	114 patients
Amiloride or hydrocnlorothiazide, mean ( ± SD)	14.7 ± 7.50	17.7 ± 3.50	17.7 ± 3.50	17.7 ± 3.50

Lipid lowering drugs	87 patients	124 patients	84 patients	84 patients
Torvastatin mean ( ± SD) dose	20.34 ± 8.76	21.34 ± 6.76	21.34 ± 6.76	21.34 ± 6.76
Simvastatin mean ( ± SD) dose	21.34 ± 7.67	22.43 ± 7.21	22.43 ± 7.21	22.43 ± 7.21
Pravastatin mean ( ± SD) dose	19.87 ± 8.67	24.54 ± 8.12	24.54 ± 8.12	24.54 ± 8.12

Nitroglycerine or analogue	126 patients	119 patients	139 patients	149 patients
Isosorbide mononitrate, mean ( ± SD) dose	36.67 ± 11.2	35.34 ± 10.56	35.34 ± 10.56	35.34 ± 10.56

Bronchodilators	59 patients	87 patients	97 patients	104 patients
Steroids, mean ( ± SD) dose	25.78 ± 11.2	31.8 ± 7.7	21.8 ± 6.5	31.8 ± 7.3

A combination of the above therapies	112 patients	183 patients	245 patients	345 patients

**Table 3:** A synopsis of the SNC drugs employed by the patients.

Drug utilization	baseline	12 months	24 months	36 months
Benzodiazepines	44 patients	89 patients	98 patients	104 patients
lorazepam, mean ( ± SD) dose	1.27 ± 0.3 mg/day	2.56 ± 0.65 mg/day	3.94 ± 1.5 mg/day	4.56 ± 1.65 mg/day
delorazepam, mean ( ± SD) dose	1.21 ± 0.8 mg/day	2.61 ± 1.29 mg/day	3.1 ± 1.54 mg/day	4.1 ± 1.89 mg/day
bromazepam, mean ( ± SD) dose	2.11 ± 1.1 mg/day	3.41 ± 0.8 mg/day	4.6 ± 1.4 mg/day	5.41 ± 1.8 mg/day

Typical neuropletics	28 patients	56 patients	98 patients	167 patients
Haloperidol, mean ( ± SD) dose	1.56 ± 0.54 mg/day	2.34 ± 0.67 mg/day	2.87 ± 1.54 mg/day	3.56 ± 0.54 mg/day
promazine chloridate, mean ( ± SD) dose	53.12 ± 12.23 mg/day	59.12 ± 16. 91 mg/day	63.12 ± 7.2 mg/day	67.12 ± 1.56 mg/day

Atypical neurolpetics	4 patients	23 patients	63 patients	53 patients
olanzapine, mean ( ± SD) dose	4.0 ± 1.10 mg/day	5.1 ± 2.34 mg/day	5.6 ± 1.6 mg/day	5.9 ± 2.94 mg/day
quetiapine, mean ( ± SD) dose	37.5 ± 5.21 mg/day	56.9 ± 3.5 mg/day	66.8 ± 3.5 mg/day	89.9 ± 3.5 mg/day

**Table 4:** Tests results in the patients observed during follow-up.

Tests	Baseline	12 months	24 months	36 months
Clinical dementia rating	1.76 ± 0.23	2.13 ± 0.11	2.84 ± 0.4	2.98 ± 0.61
Neuro psychiatric inventory	24.45 ± 7.25	41.84 ± 2.13	56.84 ± 12.1	66.03 ± 8.81
Barthel index	89.41 ± 11.3	81.14 ± 0.11	76.84 ± 6.11	61.17 ± 7.3
Instrumental activity of daily living	6.8 ± 0.34	5.84 ± 1.3	4.67 ± 1.32	3.9 ± 0.17
Hachinski ischemic score	7.6 ± 0.1	6.84 ± 0.11	6.32 ± 0.3	6.12 ± 0.12

**Table 5:** Gait tests results in the patients observed during follow-up.

Tests	Baseline	12 months	24 months	36 months
TINETTI equilibrium	10.1 ± 0.1	9.17 ± 0.7	8.27 ± 1.1	7.17 ± 1.7
TINETTI gait	11.7 ± 1.7	9.6 ± 0.9	9.2 ± 0.3	8.12 ± 0.13
TINETTI tot.score	21.8 ± 1.8	18.47 ± 1.4	17.7 ± 1.56	15.47 ± 1.6

**Table 6:** Results at 36 months: a comparison over 12 and 24 months.

Tests at 36 months	Over baseline	Over 12 months	Over 24 months
Clinical dementia rating	+1.22 ± 0.4 *P* < .01	+0.85 ± 0.2 ns	+0.14 ± 0.21 ns
NeuroPsychiatric inventory	+31. 58 ± 6.4 *P* < .01	+24.19 ± 5.9 *P* < .01	+9.19 ± 5.9 *P* < .01
Barthel index	−28.04 ± 3.4 *P* < .01	−25.17 ± 3.4 *P* < .01	−13.57 ± 3.4 *P* < .05
Instrumental activity of daily living	−2.1 ± 1.2 *P* < .05	−1.7 ± 0.3 *P* < .05	−0.4 ± 3.4 ns
Hachinski ischemic score	−1.18 ± 0.2 *P* < .05	−0.7 ± 0.3 ns	−0.2 ± 0.1 ns

**Table 7:** Results of gait scores at 36 months: a comparison over 12 and 24 months.

Tests at 36 months	Over baseline	Over 12 months	Over 24 months
TINETTI equilibrium	−3.1 ± 0.6 *P* < .01	−2 ± 1.01 *P* < .01	−1.1. ± 0.6 *P* < .05
TINETTI gait	−3.5 ± 1.4 *P* < .01	−1.5 ± 0.7 *P* < .05	−1.1. ± 0.1 *P* < .05
TINETTI tot.score	−6.47 ± 0.2 *P* < .01	−3 ± 0.1 *P* < .05	−2.3 ± 0.4 *P* < .05
